# Fosfomycin in Complicated Intra-Abdominal Infections in an Intensive Care Setting: Does It Improve the Outcome? A Retrospective Observational Study

**DOI:** 10.3390/antibiotics14111104

**Published:** 2025-11-02

**Authors:** Giovanni Genga, Federico Ragni, Maria Carolina Benvenuto, Elisabetta Svizzeretto, Andrea Tommasi, Giuseppe Vittorio Luigi De Socio, Daniela Francisci, Carlo Pallotto

**Affiliations:** 1 Infectious Diseases Clinica, Santa Maria della Misericordia Hospital, University of Perugia, 06100 Perugia, Italy; 2 Infectious Diseases Unit, Santa Maria Hospital, University of Perugia, 06123 Terni, Italy

**Keywords:** fosfomycin, intra-abdominal infection, IAI, source control, intensive care

## Abstract

**Background**: Intra-abdominal infection (IAI) is a challenging condition that needs both medical and surgical treatment and it is still associated with high morbidity and mortality rates. Fosfomycin is approved for use in combination therapy for IAIs. The aim of this study was to evaluate the impact of intravenous fosfomycin addition in a combination regimen for IAI treatment in an intensive care setting. **Methods**: We performed a retrospective, observational, monocentric study. We enrolled patients admitted to the ICU with IAIs from April 2022 to June 2024. Patients were divided into two groups: Group A, standard treatment; and Group B, combination therapy including fosfomycin. Primary endpoints were clinical response at 7 days and in-hospital mortality; moreover, a risk factor analysis for mortality was also performed. **Results**: In total, 104 patients were enrolled, 85 in Group A, and 19 in Group B. Groups were homogenous in regard to demographics, but clinical condition was slightly worst in Group B. Source control < 24 h was performed in 69.6% and 33.3% cases in Group A and Group B, respectively (*p* = 0.017). Clinical response on day 7 (81.2% vs. 73.7%, *p* = 0.675) and in-hospital mortality (27.1% vs. 47.2%, *p* = 0.145) were comparable. Univariate and multivariate analysis highlighted Charlson Comorbidity Index (CCI) (*p* = 0.04) and septic shock (*p* = 0.029) as risk factors, and effective empirical therapy (*p* = 0.04) as the protective factor; fosfomycin was not directly associated with outcome improvement. **Conclusions**: The outcome was comparable between groups; clinicians preferred to administer a combination regimen including fosfomycin in patients with statistically significant greater severity of illness and without early source control.

## 1. Introduction

Intra-abdominal infections (IAIs) were defined by the Centers for Disease Control and Prevention (CDC) as an infectious process affecting the pancreas, gallbladder, bile ducts, liver (excluding viral hepatitis), spleen, peritoneum, retroperitoneal, subdiaphragmatic space, or other intra-abdominal tissue [1]. Clinically, IAIs are classified as complicated (cIAI) when the infective process is extended beyond the hollow viscus of origin, leading to peritonitis and possible abscess formation [2]. IAIs currently represent the third leading global cause of death for infective diseases, behind lower-respiratory-tract infections and bloodstream infections [3]. The literature data estimate a mortality rate of approximately 10% for complicated intra-abdominal infections [4], and this rate may be as high as 30% in critically ill patients admitted to the intensive care unit [5], where cIAI management could often require surgical treatment [6]. Fosfomycin is an “old” antibiotic that was discovered in 1969 in Spain, isolated from cultures of *Streptomyces fradiae*, *Streptomyces viridochromogenes*, and *Streptomyces wedmorensis* [7]; it is characterized by its activity against both Gram-positive and Gram-negative bacteria. It was approved for the treatment of cIAI and other types of infections, mostly in combination therapy regimens. Several studies have evaluated the role of fosfomycin as part of a combination therapy in infections due to “difficult to treat” (DTT) and “multi-drug-resistant” (MDR) bacteria [8]. A peculiar aspect of fosfomycin is its capability to consistently penetrate—reaching effective concentration—in several tissues and body districts, including the biliary tract, gallbladder, and ascitic fluid and abscess cavities [9,10]. This study aimed to evaluate the impact of fosfomycin addition in combination therapy in patients admitted to an intensive care setting for cIAI.

## 2. Materials and Methods

This was an observational, retrospective, monocentric study. We enrolled all the patients admitted to the intensive care unit (ICU) of our University Hospital with a diagnosis of IAI from April 2022 to June 2024. Our exclusion criteria were as follows: (i) exitus within 48 h of antibiotic therapy initiation, (ii) pregnancy, and (iii) refusal to provide informed consent. Patients were divided into two groups according to fosfomycin administration as follows: Group A consisted of patients for whom fosfomycin was not administered, while Gorup B consisted of patients treated with an antibiotic regimen that included fosfomycin. We considered only cases in which fosfomycin administration lasted for at least 72 h. All antibiotic treatments were chosen by an infectious-disease consultant in agreement with colleagues of the intensive care unit and were based both on clinical and laboratory data and on microbiologic findings (antibiogram of isolated microorganisms and local epidemiology). In particular, in culture-positive cases, fosfomycin administration was continued only when the isolate resulted as susceptible to this drug; meanwhile, in culture-negative cases, the treatment choice was based on local epidemiology and patients’ risk factors. The primary endpoint of the present study was positive clinical outcome on day 7 of treatment. Positive outcome was defined as clinical improvement. Source control was defined as an intervention to reduce bacterial load (surgery, debridement, and drainage); it was evaluated within 24 h and within 48 h from clinical emergence of sign and/or symptoms of IAI. We consider cases of hypernatremia to be at least 2 consecutive natremia values > 145 mEq/L or 1 value > 150 mEq/L in patients with a previously normal natremia.

The groups were compared in terms of the following: (i) demographic characteristics (age and gender); (ii) clinical scores (Charlson Comorbidity Index (CCI), Apache II score, and SOFA score); (iii) clinical response at 7 days; (iv) laboratory values pre- and post-antibiotic therapy; (v) percentage of hospital and community infections; (vi) etiology (when available), along with genotypic and phenotypic antibiotic-resistance profile; (vii) evolution toward sepsis and septic shock; (viii) blood cultures; (ix) empirical and targeted antibiotic therapy, including possible antifungal therapy; (x) execution of source control within 24 h or within 48 h or later; (xi) in-hospital mortality distinguishing non-infectious causes; and (xii) mortality at 30 and 90 days. Exclusively for Group B, the dosage of fosfomycin and the possible interruption of treatment due to adverse reactions (In particular hypernatremia) were evaluated.

A descriptive statistical analysis was conducted for the study population, along with an inferential analysis for the two groups in which continuous and discrete variables were compared using parametric and non-parametric tests, as appropriate. In particular, continuous variables with normal distribution were described as mean and standard deviation (SD), while non-continuous variables were described as median and interquartile range (IQR). Student’s *t*-test and the Mann–Whitney U test were used to compare means and medians, respectively. Frequencies were described as percentages and compared with chi-squared test, along with Yates’ correction, when appropriate. Univariate and multivariate analyses using logistic regression were performed to assess risk factors for treatment failure, considering the primary outcome as clinical response at 7 days. Variables that reached a *p*-value < 0.1 at univariate analysis were included in the multivariate analysis, while mixed variables were excluded. Effect size was calculated only for variables with *p* < 0.1. Cohen’s D test was used for means, Cramer’s V test was used for frequencies, and, for medians, a Cramer’s V test was performed on a contingency table obtained with Mood’s median test. We interpreted Cohen’s D test as follows: <0.2, 0.2–0.5, 0.5–0.8, and >0.8 as negligible, small, medium, and large effect, respectively. Cramer’s V test for 1 degree of freedom was interpreted as follows: <0.1, 0.1–0.3, 0.3–0.5, and >0.5 as negligible, small, medium, and large effect size. In general, a *p*-value <0.05 was considered to be statistically significant.

In our post hoc analysis, we focused on patients with septic shock, and for all of those patients, the same descriptive and inferential analyses were conducted. In addition to this, again, for patients with septic shock, both a univariate and multivariate analysis using logistic regression were performed to assess risk factors for clinical non-response at seven days. Variables that reached a *p*-value <0.1 were included in the multivariate analysis, while mixed variables were excluded.

## 3. Results

We evaluated 124 patients with IAI; 20 patients were excluded according to the exclusion criteria, so the whole study population consisted of 104 patients. In total, 85 patients were included in Group A, and 19 in Group B. Table 1 reports the characteristics of the whole study population and Group A and Group B.

The two groups were considered to be homogenous in terms of demographic characteristics and clinical conditions, even if Group B showed higher clinical-score values (CCI 6 vs. 5, *p* = 0.347; APACHE 2 score, 13.5 vs. 11, *p* = 0.201; and SOFA score, 3.5 vs. 3, *p* = 0.267 in Group A and Group B, respectively) and a higher percentage of septic shock (73.7% vs. 52.9%, *p* = 0.163); these differences are not statistically significant. On the other hand, clinical management was significantly different. In Group A, source control was performed within 24 h for 69.6% of patients, while it was performed within 24 h for 33.3% patients in Group B (*p* = 0.017), and the combination of effective empirical therapy with source control within 24 h occurred in 55.7% and 14.3% of patients in Group A and Group B, respectively (*p* = 0.01). Even considering the combination of effective empirical treatment with source control within 48 h, the difference between the two groups remained statistically significant (Group A at 57% vs. Group B at 14.3%, *p* = 0.008). The effect sizes for these variables were found to be small. Nevertheless, no statistically significant differences emerged between Group A and Group B in terms of clinical response at 7 days, in-hospital mortality, mortality at 30 days, and mortality at 90 days. Among the 19 patients in Group B, 13 (68.4%) received 4 g of fosfomycin every 6 h; 3 (15.7%) received 4 g every 8 h; and the 3 last patients received 6 g every 8 h, 2 g every 6 h, and 2 g every 12 h each, respectively. We assessed the incidence of hypernatremia in Group B that led to the discontinuation of fosfomycin treatment, and the incidence was found to be 26.3% (5/19 patients); fosfomycin discontinuation occurred after a median time of 10 days (range 4–13 days). Concomitant piperacillin/tazobactam administration was described in 3/5 (60%) of patients with hypernatremia. The administration of penicillin, formulated as a sodium salt, likely contributed to the observed electrolyte imbalance in conjunction with fosfomycin.

Patients enrolled in the study were also stratified based on the endpoint “clinical response at 7 days” (if present, “positive outcome”; and if absent, “negative outcome”). In total, 83 patients had a positive outcome, while the number of patients with a negative outcome was 21. Table 2 shows the characteristics of the two groups. The APACHE II scores were higher in the negative-outcome group (11 [IQR 8–15] vs. (13.5 [12.25–16], *p* = 0.022; small effect size according to Cohen’s D test), while effective empirical therapy alone or with source control in 24 or 48 h was detected more frequently in the positive outcome group (84.3% vs. 47.6% with *p* = 0.001; 56.8% vs. 21.1% with *p* = 0.012 and 56.8% vs. 26.3% with *p* = 0.035, respectively). The effect size of septic shock and effective empirical therapy was determined to be medium.

Fosfomycin prescription was not found to be a statistically significant variable in determining outcome, according to this analysis.

Both a univariate and multivariate analysis using logistic regression were performed to assess risk factors for clinical failure at seven days. The results are shown in Table 3. The univariate analysis confirmed the negative prognostic role of septic shock (*p* = 0.03) and the protective role of the prescription of effective empirical antibiotic therapy (*p* < 0.01). The multivariate analysis consolidated the results obtained regarding septic shock (*p* = 0.029) and the prescription of effective empirical antibiotic therapy (*p* = 0.04); furthermore, it underlined a negative prognostic role of the CCI (*p* = 0.04).

Most patients receiving fosfomycin (14/19 patients, 73.7%) presented with septic shock. To better define the impact of fosfomycin on outcome, we repeated the analysis considering the subpopulation of patients with septic shock of both Group A and Group B. There are 45 patients with septic shock in Group A, while there are 14 in Group B. Also, in this case, the studied population was found to be homogeneous in terms of demographic characteristics and clinical conditions. Table 4 reports the characteristics of the study population and the relative proportions between Group A and Group B.

No statistically significant differences emerged in clinical management, although it can be noted that in Group A, effective empirical antibiotic therapy and source control within 24 h were performed in 50% of cases (21/42), while in Group B, they were performed in 18.2% of cases (2/11). No statistically significant differences were detected in terms of in-hospital mortality (*p* = 0.2), 30-day mortality (*p* = 0.626), or 90-day mortality (*p* = 0.502). Subsequently, we stratified the septic shock patients based on the endpoint “clinical response at 7 days”. The number of patients with a positive outcome was 39, while the number of patients with a negative outcome was 20. Table 5 shows the differences between the two groups.

The results obtained show a statistically significant difference in the mean age (*p* = 0.043) and in the prescription of effective empirical antibiotic therapy (*p* = 0.009), in addition to the strong correlation between the lack of clinical response at 7 days and in-hospital mortality (*p* < 0.001), and mortality at 30 days (*p* < 0.001) and at 90 days (*p* < 0.001). Both a univariate and multivariate analysis using logistic regression were performed to assess risk factors for clinical failure at seven days for patients with septic shock. The results are shown in Table 6.

The univariate analysis highlights the negative prognostic role of median age (*p* = 0.046), the protective role of the prescription of effective empirical antibiotic therapy (*p* = 0.05), and its association with source control within 24 h (*p* = 0.031). The multivariate analysis confirmed only the protective role of effective empirical antibiotic therapy (*p* = 0.013).

## 4. Discussion

Elevated morbidity and mortality are still associated with cIAI, as well as long hospital stays. The management of this kind of infection could be difficult, and it requires a specific expertise, especially because of the necessary combined medical and surgical approach [4,11,12]. In this study, we aimed to explore the possible impact of the addition of fosfomycin in a combination antibiotic regimen on patients’ outcome. Fosfomycin is a relatively small molecule that is capable of effectively penetrating several districts, including the peritoneal cavity [8,9,10], and it was approved for the treatment of cIAI. Moreover, it is easy to handle and showed an elevated possibility to be synergistic with several other anti-Gram-positive and anti-Gram-negative antibiotics even against multidrug-resistant organisms [12,13,14].

In our study, in-hospital mortality in the whole study population was about 30%. This finding is consistent with the literature, where poor outcome was described in 23–38% of cases [15,16]. We did not detect a statistically significant difference between Group A and Group B, but several factors could explain this finding and justify fosfomycin administration as an adjunctive therapy.

Clinical presentations between Group A and Group B were comparable in terms of statistical significance; however, we observed that patients of Group B seem to be more compromised, as highlighted by all the evaluated scores (APACHE II, SOFA, and CCI), as well as the higher incidence of septic shock. In Group A, infections complicated by bacteraemia were 25.3%, which is a lower percentage, though not statistically significant, compared to Group B, where such infections occurred in 38.9% of cases. The elevated percentage of critical patients with septic shock—expected data in the intensive care setting in which this study was performed—could be a crucial element. As stated by Sartelli and colleagues [11], critical patients were generally under-represented in cIAI trials, especially when a new treatment strategy or drug was investigated. This sort of selection could lead to a partial misinterpretation of data. On the other hand, our findings might be considered, from this point of view, as closer to the real world.

Source control represents a cornerstone in cIAI, and its timing is fundamental [16]. In our study, an early source control, within 24 h, was significantly more frequent in Group A (69.6% vs. Group B at 33.3%). Patients treated with fosfomycin (Group B), despite more severe clinical conditions, underwent source control within 24 h less frequently, and it influenced patients’ outcome indeed. We also evaluated the impact of the combination of source control (within 24 or 48 h) and effective antibiotic treatment with similar findings. Considering the pivotal role of early source control in the management of intra-abdominal infections, it can be stated that Group A patients received more adequate medical and surgical management compared to Group B patients. Despite the aforementioned findings, no statistically significant differences in the clinical outcomes at 7 days were observed between the two groups.

In-hospital mortality rates at 30 and 90 days were higher in Group B, but no statistically significant differences were observed in comparison with Group A. Overall mortality rates observed both in groups A and B and in the entire cohort are in line with the data available in the scientific literature. A recent review published by Napolitano et al. in 2022 [17] highlights a mortality rate for IAIs in ICU ranging widely from 5 to 50%. The outcomes and results were strongly influenced by appropriate antimicrobial therapy, effective source control, and variables such as patient comorbidities and severity of illness. The findings of the present study—showing that septic shock and inadequate empirical antimicrobial therapy represent risk factors for unfavorable outcomes in cIAI—are consistent with those reported in the previous literature [18,19]. After stratifying Group A and Group B patients for septic shock and comparing them, we observed that patients in Group B who underwent source control within 24 to 48 h had lower rates compared to Group A, respectively, i.e., 50% vs. 18.2%. This difference is not statistically significant (*p* = 0.059); however, it seems to highlight a trend toward better clinical management and surgical outcomes in Group A. These differences were not statistically significant. Results obtained in the population with septic shock confirmed at multivariate analysis the adequate antimicrobial empiric therapy as a protective factor for clinical response, in accordance with numerous studies from the scientific literature [20]. The use of fosfomycin was not found to be statistically significant in determining a favorable outcome, either in the overall population or in the subgroup of patients with septic shock. The differences observed between Group A and Group B seem to suggest that clinicians preferred to administer combination regimens including fosfomycin in patients with greater severity of illness and without early source control. Therefore, further studies evaluating the use of intravenous fosfomycin in patients undergoing more homogeneous surgical management may yield different results and are warranted.

The incidence of hypernatremia was observed in 26.3% (5/19) of patients in Group B. In 60% (3/5) of these cases, piperacillin–tazobactam was administered in combination with fosfomycin. It is likely that the sodium salt formulation of piperacillin–tazobactam, together with fosfomycin, contributed to the development of hypernatremia. The latest IDSA guidelines for complicated urinary tract infections, published in 2025, report a sodium content of 330 mg per gram of IV fosfomycin disodium and 65 mg per gram of piperacillin–tazobactam [21]. It is therefore important to bear in mind that IV fosfomycin can exacerbate cardiac failure among individuals unable to tolerate large increases in volume [21,22,23].

This study has some limitations, such as its monocentric and retrospective design. As it is a retrospective study, we cannot exclude the influence on results from unrecognized variables with consequent potential effects on outcome. Antibiotic treatment schemes were chosen by an infectious-disease clinician after considering clinical and laboratory conditions and personal opinion; consistently with the retrospective design, this could represent a bias, especially in a limited-sample-size study like this one. Moreover, the limited sample size itself, especially for Group B, was an additional limitation, and, as a consequence, the effect size of significantly different variables was small or medium.

In conclusion, we observed a similar mortality rate and clinical response after 7 days in the two groups; however, clinical condition at presentation and management were both better in Group A. Therefore, we can hypothesize that fosfomycin could have a role in making the outcome measures comparable. Further studies comparing therapeutic regimens with or without fosfomycin are needed to clarify the role of this antibiotic in the treatment of severe infections, including cIAI and those due to multidrug-resistant bacteria.

## Figures and Tables

**Table 1:** Characteristics of the whole study population, Group A, and Group B.

	Total of Patients (n = 104)	Group A (n = 85)	Group B (n = 19)	*p*-Value	Effect Size
Age, median (IQR)	72 (61.75–80.25)	71 (60–80)	74 (71–81)	0.258	
Males, n (%)	61 (58.7)	48 (56.5)	13 (68.4)	0.485	
CCI, median (IQR)	5 (3–7)	5 (3–7)	6 (4.5–8)	0.347	
APACHE II, median (IQR)	11 (8–15)	11 (8–15)	13.5 (11–18.25)	0.201	
SOFA, median (IQR)	3 (2–5)	3 (2–4)	3.5 (2–6.75)	0.267	
Septic shock, n (%)	59 (56.7)	45 (52.9)	14 (73.7)	0.163	
Hospital-acquired Infections, n (%)	41 (39.4)	32 (37.6)	9 (47.4)	0.6	
Infections with bacteremia, n (%)	26/93 (28)	19/75 (25.3)	7/18 (38.9)	0.391	
Polymicrobial infections, n (%)	50/77 (64.9)	43/64 (67.2)	7/13 (53.8)	0.548	
Infections due to non-wild-type microorganisms, n (%)	33/77 (42.9)	27/64 (42.2)	6/13 (46.1)	0.965	
Fungal etiology, n (%)	23 (22.1)	20 (23.5)	3 (15.8)	0.668	
PCT on admission ng/mL, mean (SD)	29.86 (36.97)	29.79 (36.94)	30.17 (38.1)	0.968	
Source control < 24 h, n (%)	60/94 (63.8)	55/79 (69.6)	5/15 (33.3)	**0.017**	**0.25**
Source control < 48 h, n (%)	64/94 (68.1)	55/79 (69.6)	9/15 (60)	0.667	
Source control, n (%)	82/94 (87.2)	70/79 (88.6)	12/15 (80)	0.621	
Antifungal therapy, n (%)	75 (72.1)	63 (74.1)	12 (63.2)	0.496	
effective empirical therapy, n (%)	80 (76.9)	67 (78.8)	13 (68.4)	0.502	
Effective empirical therapy + source control < 24 h, n (%)	46/93 (49.5)	44/79 (55.7)	2/14 (14.3)	**0.01**	**0.27**
Effective empirical therapy + source control < 48 h, n (%)	47/93 (50.5)	45/79 (57)	2/14 (14.3)	**0.008**	**0.28**
Clinical response at seven days, n (%)	83 (79.8)	69 (81.2)	14 (73.7)	0.675	
In-hospital mortality, n (%)	32 (30.8)	23 (27.1)	9 (47.4)	0.145	
30-day mortality, n (%)	26 (25)	19 (22.4)	7 (36.8)	0.305	
90-day mortality. n (%)	39 (37.5)	29 (34.1)	10 (52.6)	0.213	

Abbreviations: IQR, interquartile range; CCI, Charlson Comorbidity Index; SD, standard deviation.

**Table 2:** Characteristics of the study population after stratification per outcome.

	Positive Outcome (n = 83)	Negative Outcome (n = 21)	*p*-Value	Effect Size *
Age, median (IQR)	71 (60–80)	77 (68–82)	0.168	
Males, n (%)	50 (60.2)	11 (52.4)	0.685	
CCI, median (IQR)	5 (3–7)	6 (5–9)	0.159	
APACHE, median (IQR)	11 (8–15)	13.5 (12.25–16)	**0.022**	0.31
SOFA, median (IQR)	3 (2–4)	3 (3–5.5)	0.211	
Septic shock, n (%)	39 (47)	20 (95.2)	**<0.001**	0.37
Hospital-acquired infections, n (%)	31 (37.3)	10 (47.6)	0.541	
Infections with bacteremia, n (%)	18/75 (24)	8/18 (44.4)	0.149	
Polymicrobial infections, n (%)	40/62 (64.5)	10/15 (66.7)	0.885	
Fungal etiology, n (%)	18 (21.7)	5 (23.8)	0.932	
Etiology from germs characterized by antibiotic-resistance profiles, n (%)	24/62 (38.7)	9/15 (60)	0.228	
PCT on admission ng/mL, mean (SD)	29.91 (37.29)	29.68 (36.57)	0.98	
Source control < 24 h, n (%)	50/75 (66.7)	10/19 (52.6)	0.384	
Source control < 48 h, n (%)	52/75 (69.3)	12/19 (63.2)	0.81	
Source control, n (%)	67/75 (89.3)	15/19 (78.9)	0.408	
Antifungal therapy, n (%)	58 (69.9)	17 (81)	0.46	
Effective empirical therapy, n (%)	70 (84.3)	10 (47.6)	**0.001**	0.35
Effective empirical therapy + source control < 24 h, n (%)	42/74 (56.8)	4/19 (21.1)	**0.012**	0.29
Effective empirical therapy + source control < 48 h, n (%)	42/74 (56.8)	5/19 (26.3)	**0.035**	0.25
Fosfomycin, n (%)	14 (16.9)	5 (23.8)	0.675	

Abbreviations: IQR, interquartile range; CCI, Charlson Comorbidity Index; SD, standard deviation. Note: * Tests and interpretations were specified in the Materials and Methods section.

**Table 3:** Univariate and multivariate analyses for risk factors for clinical failure at 7 days.

	Univariate OR (95%CI)	*p*-Value	Multivariate OR (95%CI)	*p*-Value
Age	1.031 (0.99–1.073)	0.141		
Male sex at birth	0.729 (0.277–1.901)	0.514		
CCI	1.152 (0.999–1.327)	0.051	1.239 (1.01–1.52)	**0.04**
APACHE score	1.05 (0.972–1.133)	0.214		
SOFA score	1.121 (0.947–1.328)	0.184		
Septic shock	22.564 (2.893–176.003)	**0.003**	11.36 (1.281–100.762)	**0.029**
Hospital acquired infections	1.525 (0.581–4.003)	0.391		
Infections with bacteremia	2.533 (0.869–7.387)	0.089	1.852 (0.509–6.743)	**0.35**
Polymicrobial infections	1.1 (0.334–3.627)	0.876		
Infections due to non-wild-type microorganisms	2.375 (0.75–7.52)	0.141		
Fungal etiology	1.129 (0.364–3.5)	0.834		
PCT on admission	1 (0.987–1.013)	0.979		
Source control < 24 h	0.556 (0.2–1.542)	0.259		
Source control < 48 h	0.758 (0.264–2.174)	0.607		
Source control	0.448 (0.119–1.684)	0.234		
Antifungal therapy	1.832 (0.56–5.997)	0.317		
Effective empirical therapy	0.169 (0.06–0.478)	**<0.001**	0.141 (0.037–0.541)	**0.004**
Effective empirical therapy + source control < 24 h	0.203 (0.062–0.671)	**0.009**		
Effective empirical therapy + source control < 48 h	0.272 (0.089–0.834)	**0.023**		
Fosfomycin	1.54 (0.484–4.898)	0.464		

Abbreviations: CCI, Charlson Comorbidity Index.

**Table 4:** Characteristics of the whole study population, Group A, and Group B (only patients with septic shock).

	Total (n = 59)	Group A (n = 45)	Group B (n = 14)	*p*-Value	Effect Size
Age, median (IQR)	72 (58.5–79)	69 (58–79)	73.5 (70.5–76.5)	0.509	
Males n (%)	36 (61)	27 (60)	9 (64.3)	0.979	
CCI, median (IQR)	5 (3.5–7)	5 (4–7)	5.5 (3.25–6.75)	0.912	
APACHE, median (IQR)	13 (10–19)	13 (9–17.25)	15 (11–22)	0.162	
SOFA, median (IQR)	4 (3–6)	4 (3–5.75)	5 (3–9)	0.337	
Hospital-acquired infections, n (%)	24 (40.7)	18 (40)	6 (42.9)	0.903	
Infections with bacteremia, n (%)	22/55 (40)	16/42 (38.1)	7/13 (53.8)	0.494	
Polymicrobial infections, n (%)	31/46 (67.4)	24/35 (68.6)	7/11 (63.6)	0.949	
Infections due to non-wild-type microorganisms, n (%)	22/46 (47.8)	17/35 (48.6)	5/11 (45.5)	0.869	
Fungal etiology, n (%)	10 (16.9)	8 (17.8)	2 (14.3)	0.917	
PCT on admission ng/mL, mean (SD)	37.82 (40.68)	38.18 (41.07)	36.66 (40.91)	0.904	
Source control < 24 h, n (%)	32/53 (60.4)	28/42 (66.7)	4/11 (36.4)	0.138	
Source control < 48 h, n (%)	34/53 (64.2)	28/42 (66.7)	6/11 (54.5)	0.694	
Source control, n (%)	44/53 (83)	34/42 (81)	10/11 (90.9)	0.74	
Antifungal therapy, n (%)	43 (72.9)	35 (77.8)	8 (57.1)	0.241	
Effective empirical therapy, n (%)	41 (69.5)	32 (71.1)	9 (64.3)	0.871	
Effective empirical therapy + source control < 24 h, n (%)	23/53 (43.4)	21/42 (50)	2/11 (18.2)	0.059	0.21
Effective empirical therapy + source control < 48 h, n (%)	23/53 (43.4)	21/42 (50)	2/11 (18.2)	0.059	0.21
Clinical response at 7 days, n (%)	39 (66.1)	29 (64.4)	10 (71.4)	0.874	
Hospital mortality, n (%)	23 (39)	15 (33.3)	8 (57.1)	0.2	
30-day mortality, n (%)	20 (33.9)	14 (31.1)	6 (42.9)	0.626	
90-day mortality, n (%)	27 (45.8)	19 (42.2)	8 (57.1)	0.502	

Abbreviations: IQR, interquartile range; CCI, Charlson Comorbidity Index; SD, standard deviation.

**Table 5:** Characteristics of the study population after stratification per outcome (only patients with septic shock).

	Positive Outcome (n = 39)	Negative Outcome (n = 20)	*p*-Value	Effect Size
Age, median (IQR)	69 (57.5–75)	75.5 (67.75–82)	**0.043**	0.27
Males, n (%)	25 (64.1)	11 (55)	0.692	
CCI, median (IQR)	5 (3–7)	5.5 (4.75–8.25)	0.19	
APACHE, median (IQR)	11 (8.75–19)	13 (12–16)	0.327	
SOFA, median (IQR)	4 (3–6)	3.5 (3–5.75)	0.841	
Hospital-acquired infections, n (%)	14 (35.9)	10 (50)	0.445	
Infections with bacteremia, n (%)	15/38 (39.5)	7/17 (41.2)	0.858	
Polymicrobial infections, n (%)	21/31 (67.7)	10/15 (66.7)	0.793	
Infections due to non-wild-type microorganisms, n (%)	13/31 (41.9)	9/15 (60)	0.404	
Fungal etiology, n (%)	5 (12.8)	5 (25)	0.416	
PCT on admission ng/mL, mean (SD)	41.24 (42.54)	31.15 (36.89)	0.372	
Source control < 24 h, n (%)	22/35 (62.9)	10/18 (55.6)	0.827	
Source control < 48 h, n (%)	22/35 (62.9)	12/18 (66.7)	0.977	
Source control, n (%)	29/35 (82.9)	15/18 (83.3)	0.732	
Antifungal therapy, n (%)	27 (69.2)	16 (80)	0.568	
Effective empirical therapy, n (%)	32 (82.1)	9 (45)	**0.009**	0.34
Effective empirical therapy + source control < 24 h, n (%)	19/35 (54.3)	4/18 (22.2)	**0.001**	**0.48**
Effective empirical therapy + source control < 48 h, n (%)	19/35 (54.3)	5/18 (27.8)	0.122	
Fosfomycin, n (%)	10 (25.6)	4 (20)	0.874	
In-hospital mortality, n (%)	8 (20.5)	15 (75)	**<0.001**	0.49
30-day mortality, n (%)	7 (17.9)	13 (65)	**<0.001**	0.43
90-day mortality, n (%)	11 (28.2)	16 (80)	**<0.001**	0.46

Abbreviations: IQR, interquartile range; CCI, Charlson Comorbidity Index; SD, standard deviation.

**Table 6:** Univariate and multivariate analysis for risk factors for clinical failure at 7 days (only patients with septic shock).

	Univariate OR (95%CI)	*p*-Value	Multivariate OR (95%CI)	*p*-Value
Age	1.051 (1.001–1.104)	**0.046**	1.03 (0.974–1.09)	0.303
Gender male	0.684 (0.228–2.051)	0.498		
CCI score	1.158 (0.973–1.379)	0.099	1.112 (0.895–1.381)	0.338
APACHE score	1.023 (0.933–1.120)	0.633		
SOFA score	0.999 (0.828–1.206)	0.994		
Hospital-acquired infections	1.786 (0.598–5.331)	0.299		
Infections with bacteremia	1.073 (0.335–3.439)	0.905		
Polymicrobial infections	0.952 (0.257–3.534)	0.942		
Infections due to non-wild-type microorganisms	2.077 (0.592–7.289)	0.254		
Fungal etiology	2.267 (0.57–9.014)	0.245		
PCT on admission	0.994 (0.980–1.007)	0.365		
Source control < 24 h	0.739 (0.233–2.345)	0.607		
Source control < 48 h	1.182 (0.357–3.908)	0.784		
Source control	1.035 (0.226–4.728)	0.965		
Antifungal therapy	1.778 (0.49–6.455)	0.382		
Effective empirical therapy	0.179 (0.054–0.595)	**0.005**	0.202 (0.057–0.711)	**0.013**
Effective empirical therapy + source control < 24 h	0.241 (0.066–0.879)	**0.031**		
Effective empirical therapy + source control < 48 h	0.324 (0.095–1.105)	0.072		
Fosfomycin	0.725 (0.196–2.688)	0.631		

Abbreviations: CCI, Charlson Comorbidity Index.

## Data Availability

The datasets analyzed during the current study are not publicly available due to privacy protection reasons but are available from the corresponding author on reasonable request.
